# Prepartum and Postpartum Feed Restrictions Affect Blood Metabolites and Hormones Reducing Colostrum and Milk Yields in Fat-Tailed Dairy Sheep

**DOI:** 10.3390/ani11051258

**Published:** 2021-04-27

**Authors:** Mousa Zarrin, Meysam Sanginabadi, Mahrokh Nouri, Amir Ahmadpour, Lorenzo E. Hernández-Castellano

**Affiliations:** 1 Department of Animal Science, Faculty of Agriculture, Yasouj University, Student Street, Yasouj 75918-74831, Iran; m.sanginabadi92@yahoo.com (M.S.); mahaknouri95@gmail.com (M.N.); ahmadpouramir@yahoo.com (A.A.); 2 Animal Production and Biotechnology Group, Institute of Animal Health and Food Safety, Universidad de Las Palmas de Gran Canaria, 35413 Arucas, Spain

**Keywords:** ewe, mammary gland, metabolism, parturition

## Abstract

**Simple Summary:**

Despite the fact that fat-tailed sheep raised for meat production are well known for being resilient to harsh environmental conditions such as pasture scarcity or low-quality feedstuffs, no studies regarding feed restriction have been performed on fat-tailed dairy sheep. In this study, prepartum feed restriction from week −5 to week −1 relative to parturition did not affect body weight. Similarly, postpartum feed restriction from week 1 to week 5 relative to parturition did not affect body weight. However, both prepartum and postpartum feed restrictions affected blood metabolites and hormones, which decreased both colostrum and milk yields postpartum.

**Abstract:**

This study aimed to investigate the effect of prepartum and postpartum feed restriction on body weight (BW), blood metabolites, and hormones as well as colostrum and milk yields and compositions in fat-tailed dairy sheep. In this study, 20 multiparous and pregnant ewes were randomly allocated to either the control (Ctrl; *n* = 10) or the feed-restricted (FR; *n* = 10) groups from week −5 to week 5 relative to parturition. Despite dry matter intake being decreased in the FR group compared to the Ctrl throughout both prepartum and postpartum periods, no differences in BW were detected between groups in any of the studied periods. Feed restriction increased both free fatty acids and beta-hydroxybutyrate concentrations during both prepartum and postpartum periods. Similarly, feed restriction increased triglyceride concentration postpartum. Additionally, feed restriction increased insulin and growth hormone and decreased prolactin concentrations during both prepartum and postpartum periods. Feed restriction caused a decreased colostrum yield and a relative increase of the main colostrum components in the FR group. Similarly, milk yield decreased in the FR group compared to the Ctrl group, although milk components were not affected. In conclusion, feed restriction did not affect BW but decreased colostrum and milk yield in fat-tailed dairy sheep.

## 1. Introduction

In developing countries, livestock systems located in tropical and sub-tropical regions are heavily dependent on natural resources (i.e., pastures). In these countries, decreased pasture availability and quality during the dry season have important consequences on the performance and health of dairy ruminants [1], especially during the transition period (i.e., from the last weeks of prepartum to the first weeks postpartum). During this period, the reduced feed intake capacity and the high demand for energy for fetal growth (i.e., prepartum) and milk production (i.e., postpartum) present an important metabolic challenge for dairy ruminants [2]. In these conditions, energy intake does not meet energy requirements for body maintenance, fetal growth, and milk production, which results in negative energy balance (NEB) and high adipose tissue mobilization [3,4]. If adaptation to NEB fails, the risk of metabolic disorders increases considerably, affecting not only animal performance but also animal health and welfare [5]. In addition, insufficient energy intake during the last weeks before parturition may affect the cell reorganization of the dry mammary gland and, therefore, affect milk yield and composition in the next lactation [6].

Fat-tailed sheep are raised in semi-arid regions of Eastern and Southern Africa, Central Asia, and numerous countries in the Middle East [7]. The common characteristic of all fat-tailed sheep is the deposition of a substantial amount of fat in the tail. Fat-tailed sheep are known for being highly resilient to harsh environmental conditions such as those related to the dry season (i.e., water scarcity and low-quality pastures and feedstuffs). According to the literature, fat depots are differently regulated in fat-tailed sheep compared to other sheep breeds during periods of feed scarcity [7,8,9]. However, most of these studies have been performed in sheep breeds used for meat production. The Lori-Bakhtiari and Turkey-Qashqai are two fat-tailed sheep breeds that are mostly used for milk production, although meat from this breed is also consumed. Consequently, both breeds are mostly considered dairy breeds. Therefore, and to the best of our knowledge, this is the first study performed regarding feed restriction in fat-tailed dairy sheep around parturition.

Based on the above-mentioned facts, the current study aimed to investigate the consequences of pre- and postpartum feed restriction on body weight (BW), blood metabolites, and hormones as well as colostrum and milk yield and composition in dairy fat-tailed ewes. This study hypothesizes that feed restriction around parturition affects BW, blood metabolites, and hormones as well as colostrum and milk yield and composition in fat-tailed sheep.

## 2. Materials and Methods

### 2.1. Animals and Management

The present study was performed at the experimental farm of Yasouj University (Yasouj, Iran). All animal procedures followed the ethical law on Animal Protection and were approved by the Committee of Animal Experiments (Yasouj University, Yasouj, Iran) under the procedure 950441007-13845. During the entire experiment, all ewes were visually healthy and had no signs of diarrhea.

This experiment used 20 multiparous and pregnant fat-tailed dairy ewes (Lori-Bakhtiari, *n* = 10; Turkey-Qashqai, *n* = 10) with an average age 40.8 ± 6.2 months and BW 56 ± 1.8 kg, which provided a power analysis = 0.85. The experimental period lasted from week −5 to week 5 relative to parturition. During the trial, all animals were kept in individual pens (1.2 × 1.0 m) located in a closed barn. Each pen was equipped with individual drink and feed containers. Animals were placed outdoor for sunlight, exercise, and hygiene operations three h twice a week after morning feeding. Three days before the expected parturition, ewes were transferred to a parturition pen (3.0 × 2.0 m) with clean straw bedding and free access to water and feed. After the first week postpartum, animals returned to the individual pens (1.2 × 1.0 m), where they stayed until the end of the experimental period (i.e., week 5 relative to parturition).

From week −7 to week −5, all animals were fed with a total mixed ration (TMR) diet formulated to fulfill 100% of the energy requirements recommended by the National Research Council [10] for dry ewes (i.e., dry diet). At week −5, animals were randomly allocated into one of the two experimental groups, including the control (Ctrl; *n* =10; Lori-Bakhtiari, *n* = 5; Turkey-Qashqai, *n* = 5) and the feed-restricted (FR; *n* = 10; Lori-Bakhtiari, *n* = 5; Turkey-Qashqai, *n* = 5) groups. From week −5 relative to parturition, ewes from the Ctrl group received the dry diet. Ewes from the FR group were fed with different energy levels from week −5 to week −1 relative to expected parturition. Thus, the FR group was fed with a diet equivalent to 100, 50, 65, 80, and 100% of the energy content of the dry diet at weeks −5, −4, −3, −2, and −1 relatives to expected parturition, respectively. After parturition, ewes from the Ctrl group were fed with a TMR diet formulated to fulfill 100% of the energy requirements recommended by the NRC [10] for early lactation ewes (i.e., early lactation diet). Similarly, the FR group received a diet equivalent to 100, 50, 65, 80, and 100% of the energy content of the early lactation diet at weeks 1, 2, 3, 4, and 5 relatives to parturition respectively. Changes in the energy content of the FR group increased progressively from 50% (week −4 and 1 relative to parturition) to 100% (week −1 and week 5 relative to parturition) to prevent rumen and metabolic acidosis. During the entire experimental period, the TMR was provided to the animals twice a day (0800 and 1700). In addition, animals had free access to drinking water and mineral blocks throughout the entire experimental period. The chemical composition and ration ingredients of the dry and early lactation diets are shown in Table 1.

### 2.2. DMI, BW, Colostrum Yield, and Milk Yield

The individual feed intake was recorded daily by weighing the offered TMR and the residual TMR of the next morning before feeding. Feed samples were collected to determine the DM of the diets and calculate dry matter intake (DMI). The dry matter (DM) content of the diets was determined according to the method described by the Association of Official Analytical Chemists (procedure 934.01; [11]). Individual BW was recorded weekly. Colostrum yield was estimated using the weight–suckling–weight method described by Benson et al. [12] with slight modifications. Briefly, all lambs were kept separate from dams and weighed before and after suckling. Then, the weight difference was added to the weight of the remaining colostrum once it was hand-milked in order to calculate the total colostrum yield. Similarly, to estimate the individual milk yield, lambs were weighed before and after milk feeding. Then, the weight difference was added to the weight of the remaining milk once it was hand-milked.

### 2.3. Colostrum, Milk, and Blood Collection

Colostrum samples (50 mL) were collected immediately at parturition. In addition, milk samples (50 mL) were collected at 0800 and 1600 twice a week during the experimental period. Both colostrum and milk samples were treated with Bronopol tablets (Broad Spectrum Microtabs, Norwood, MA, USA) and then stored at −20 °C.

During the entire experimental period (from week −5 to week 5), blood samples were collected weekly from the jugular vein using heparinized vacuum tubes (6 mL) at 0730. All blood samples were kept in wet ice and then centrifuged at 3000× *g* for 20 min at 4 °C (Hemle Labortechnik GmbH, Wehingen, Germany). Then, the plasma was aliquoted (1.5 mL) and stored at −20 °C.

### 2.4. Variables Measured in Colostrum and Milk

Colostrum and milk samples were analyzed for fat, protein, lactose, solids not fat (SNF) content, and density using a Lactoscan milk analyzer (Lactoscan S standard 1040, Basic Models, Nova Zagora, Bulgaria). Fat-corrected milk (FCM; 6%) as well as milk energy content (EVL; KJ/kg) were calculated based on the following equations as described by Milis [13], and then, EVL was converted to Mcal/kg:

FCM (% 6, kg/d) = L (kg/d) × [0.472 + 0.0088 × F (g/kg)]

EVL (Mcal/kg) = 39 × F + 18.2 × SNF + 52.

### 2.5. Variables Measured in Plasma

The plasma concentration of glucose (#1500017), triglycerides (TG; #1500032), cholesterol (#1500010), total protein (TP; #1500028), albumin (#101500), urea (#1400029), creatinine (#1400009) as well as the plasma activity of lactate dehydrogenase (LDH; #122400), alanine aminotransferase (GPT; # 1400019), and aspartate transaminase (GOT; #1400018) were determined using an automatic chemical analyzer and commercial kits (Pars Azmoon, Karaj, Iran). The intra-assay coefficients of variation were 1.5, 1.6, 0.9, 0.9, 1.3, 3.3, 3.2, 2.5, 3.6, and 2.4%, respectively. The inter-assay coefficients of variation were 0.9, 1.2, 1.1, 1.3, 1.5, 4.1, 1.8, 1.7, 1.9, and 2.2%, respectively.

Plasma free fatty acids (FFA; # FA115) and beta-hydroxybutyrate (BHB; #RB1007) concentrations were measured using commercial kits (Randox Laboratories Ltd., Crumlin, UK) following the manufacturer’s instructions. The intra-assay coefficients of variation were 4.4 and 5.1%, respectively. The inter-assay coefficients of variation were 4.7 and 37%, respectively. Insulin (# CSB-E17044Sh), growth hormone (GH; # CSB-EL009407Sh), prolactin (# CSB-E13161Sh), and progesterone (#CSB-E13176Sh) concentrations were determined using commercial ELISA kits (Cusabio, Houston, TX, USA) following the manufacturer’s instructions. All ELISA kits used in this study were validated for being used in sheep plasma. The intra-assay coefficients of variation were 6.7, 5.6, 5.3, and 4.9%, respectively. The inter-assay coefficients of variation were 12.6, 15.2, 14.7, and 12.2%, respectively.

### 2.6. Statistical Analysis

The data were tested for normal distribution using the UNIVARIATE procedure of SAS (Version 9.4, SAS Institute Inc., Cary, NC, USA). Two datasets were created based on week relative to parturition. The prepartum dataset included all data collected from week −5 to week −1. Similarly, the postpartum dataset included all data collected from week 1 to week 5. Both datasets were evaluated using the MIXED procedure of SAS. The model included feed restriction (Ctrl and FR), time (either from week −5 to week −1 or from week 1 to week 5), and the interaction (feed restriction × time) as fixed effects. Breed (Lori-Bakhtiari and Turkey-Qashqai) was set as a random effect, and the individual ewe was set as a repeated subject. Significant effects were considered when *p* ≤ 0.05. Results are presented as least squares means (LSM) ± standard error of the mean (SEM).

## 3. Results

In the present study, none of the animals showed dystocia at parturition and did not show symptoms related to metritis and mastitis during the entire experimental period. Pregnancy length was 151 ± 1.13 and 152 ± 1.12 days in the Ctrl and FR group, respectively (data shown as mean ± standard deviation). In this study, all ewes gave birth to a single lamb. No differences in birth BW were detected between singleton lambs born from the FR group and those born from the Ctrl group (4.35 ± 0.36 and 4.80 ± 0.37 kg BW, respectively; *p* > 0.05). From week 1 to week 5, all animals (i.e., ewes and lambs) were visually healthy.

### 3.1. DMI and BW

**Figure 1:**
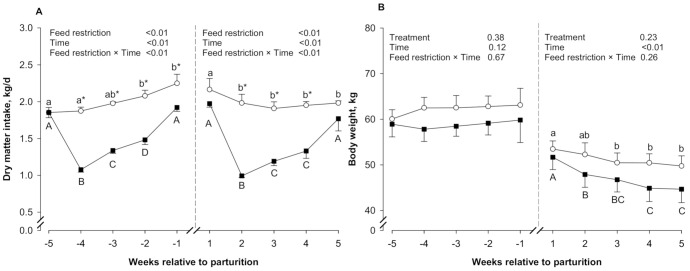
DMI (**A**) and BW (**B**) in control ewes (Ctrl, *n* = 10; ο) and feed-restricted ewes (FR, *n* = 10; ■), during both prepartum (week −5 to week −1) and postpartum (week 1 to week 5) periods. Different lowercase letters (a,b) indicate significant differences (*p* ≤ 0.05) between time points within the Ctrl group. Different uppercase letters (A–D) indicate significant differences (*p* ≤ 0.05) between time points within the FR group. * Indicates a significant difference (*p* ≤ 0.05) between the control and FR groups within each time point. Results are expressed as least square means ± standard error of the mean.

As shown in Figure 1A, an interaction between feed restriction and time was detected for DMI during both prepartum and postpartum periods (*p* ≤ 0.05). The induced feed restriction decreased DMI in the FR group compared to the Ctrl group during both prepartum and postpartum periods (*p* ≤ 0.05). As expected, no differences in DMI were detected between the Ctrl and FR groups at week −5 relative to parturition (*p* > 0.05). However, DMI was lower in the FR group than in the Ctrl group at weeks −4, −3, −2, and −1 relative to parturition (*p* ≤ 0.05). During the postpartum period, no differences in DMI were observed between groups at week 1 and week 5 postpartum (*p* > 0.05). However, DMI was lower in the FR group than in the Ctrl group at weeks 2, 3, and 4 postpartum (*p* ≤ 0.05).

As observed in Figure 1B, neither feed restriction nor time prepartum affected BW (*p* > 0.05). After parturition, BW decreased continuously in both the Ctrl and FR groups until the end of the postpartum period (*p* ≤ 0.05), although no differences were detected between groups (*p* > 0.05).

### 3.2. Blood Metabolites

Table 2 shows the blood metabolites concentrations of the Ctrl and FR groups during both prepartum and postpartum periods. In the present study, glucose, TP, urea, albumin, creatinine, cholesterol, GOT, GPT, and LDH were not affected by either feed restriction or time during either the prepartum or the postpartum periods (*p* > 0.05).

**Figure 2:**
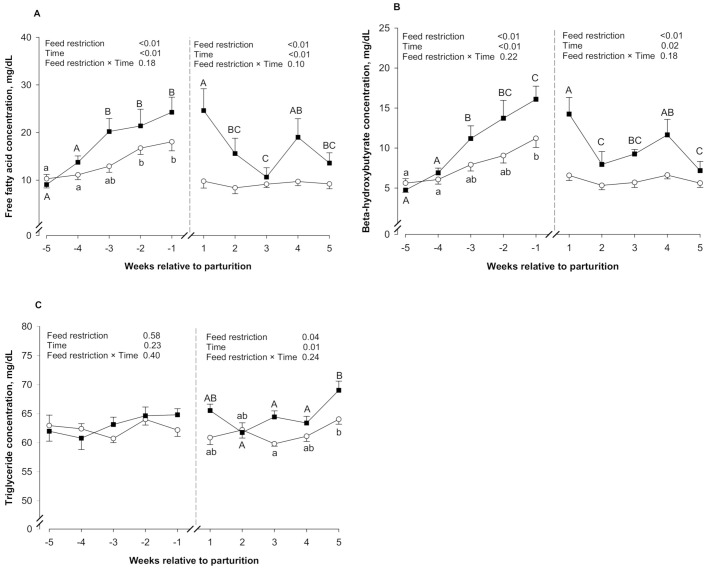
Plasma free fatty acids (**A**), beta-hydroxybutyrate (**B**), and triglycerides (**C**) in control (Ctrl, *n* = 10; ο) and feed-restricted ewes (FR, *n* = 10; ■), during both prepartum (week −5 to week −1) and postpartum (week 1 to week 5) periods. Different lowercase letters (a,b) indicate significant differences (*p* ≤ 0.05) between time points within the Ctrl group. Different uppercase letters (A–C) indicate significant differences (*p* ≤ 0.05) between time points within the FR group. Results are expressed as least square means ± standard error of the mean.

During both prepartum and postpartum periods, FFA concentrations (Figure 2A) were affected by feed restriction (*p* ≤ 0.05) and time (*p* ≤ 0.05). In both periods, FFA concentrations were higher in the FR group than in the Ctrl group (*p* ≤ 0.05). During the prepartum period, both groups had increased FFA concentrations (*p* ≤ 0.05). During the postpartum period, FFA concentrations remained constant in the Ctrl group (*p* > 0.05). In contrast, the FR group had decreased FFA concentrations from week 1 to week 3, which was followed by a slight increase at week 4 and a decrease at week 5 (*p* ≤ 0.05).

During both prepartum and postpartum periods, BHB concentrations (Figure 2B) were affected by feed restriction (*p* ≤ 0.05) and time (*p* ≤ 0.05). During the prepartum period, BHB concentrations were higher in the FR group than in the Ctrl group (*p* ≤ 0.05). Both groups had increased BHB concentrations from week −5 to week −1 during the prepartum period (*p* ≤ 0.05). In the postpartum period, BHB concentrations were higher in the FR group than the Ctrl group (*p* ≤ 0.05). During the postpartum period, BHB concentrations remained constant in the Ctrl group (*p* > 0.05). In contrast, the FR group had decreased BHB concentrations from week 1 to week 2 followed by a slight increase from week 2 to week 4 when FFA concentrations decreased again until week 5 (*p* ≤ 0.05).

During the prepartum period, TC concentrations (Figure 2C) were not affected by either feed restriction (*p* > 0.05) or time (*p* > 0.05). During the postpartum period, TG concentrations were affected by feed restriction (*p* ≤ 0.05) and time (*p* ≤ 0.05). Triglyceride concentrations were higher in the FR group than in the Ctrl group (*p* ≤ 0.05). In both groups, TG concentrations remained constant from week 1 to week 4 and increased in week 5 (*p* ≤ 0.05).

### 3.3. Blood Hormones

**Figure 3:**
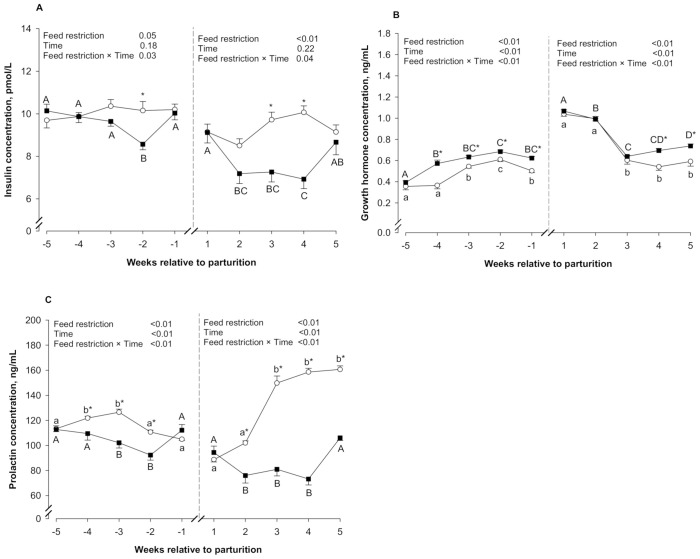
Plasma insulin (**A**), growth hormone (**B**), and prolactin (**C**) in control (Ctrl, *n* = 10; ο) and feed-restricted ewes (FR, *n* = 10; ■), during both prepartum (week −5 to week −1) and postpartum (week 1 to week 5) periods. Different lowercase letters (a,b) indicate significant differences (*p* ≤ 0.05) between time points within the Ctrl group. Different uppercase letters (A–D) indicate significant differences (*p* ≤ 0.05) between time points within the FR group. * Indicates a significant difference (*p* ≤ 0.05) between the control and FR groups within each time point. Results are expressed as least square means ± standard error of the mean.

Table 3 shows the blood hormone concentrations of the Ctrl and FR groups during both prepartum and postpartum periods. An interaction between feed restriction and time was detected for insulin concentrations (Figure 3A) during both prepartum and postpartum periods (*p* ≤ 0.05). During the prepartum period, insulin concentrations were lower in the FR group compared to the Ctrl group in week −2 (*p* ≤ 0.05). No differences were detected between groups during the rest of the prepartum period (*p* > 0.05). During the postpartum period, the FR group showed lower insulin concentrations than the Ctrl group at weeks 3 and 4 (*p* ≤ 0.05). No differences between groups were detected at weeks 1, 2, and 5 (*p* > 0.05).

An interaction between feed restriction and time was detected for GH concentrations (Figure 3B) during both prepartum and postpartum periods (*p* ≤ 0.05). During the prepartum period, no differences between groups were detected at week −5 (*p* > 0.05). However, the FR group showed higher GH concentrations than the Ctrl group during the rest of the prepartum period (*p* ≤ 0.05). During the postpartum period, no differences were detected between groups at weeks 1, 2, and 3 (*p* > 0.05). However, the FR group showed higher GH concentrations than the Ctrl group at weeks 4 and 5 relative to parturition (*p* ≤ 0.05).

An interaction between feed restriction and time was detected for prolactin concentrations (Figure 3C) during both the prepartum and postpartum periods (*p* ≤ 0.05). During the prepartum period, no differences were detected between groups at weeks −5 and −1 (*p* > 0.05). However, the FR group showed lower prolactin concentrations than the Ctrl group at weeks −4, −3, and −2 (*p* ≤ 0.05). During the postpartum period, prolactin concentrations were similar between groups at week 1 (*p* > 0.05). However, the FR group showed lower prolactin concentrations than the Ctrl group during the rest of the postpartum period (*p* > 0.05).

Progesterone concentrations were only affected by the time during the prepartum period (*p* ≤ 0.05). During that period, progesterone concentrations decreased progressively from week −5 (4.21 ± 0.22 and 4.03 ± 0.44 ng/mL in the Ctrl and FR groups, respectively) to week −1 (2.42 ± 0.43 and 2.24 ± 0.23 ng/mL in the Ctrl and FR groups, respectively) (*p* ≤ 0.05). During the postpartum period, progesterone concentrations were not affected by either feed restriction (*p* > 0.05) or time (*p* > 0.05).

### 3.4. Colostrum Yield and Composition

As described in Table 4, feed restriction reduced colostrum yield in the FR group compared to the Ctrl group (*p* ≤ 0.05). Fat, lactose, protein, and SNF percentages in colostrum were higher in the FR group than in the Ctrl group (*p* ≤ 0.05).

### 3.5. Milk Yield and Composition

Milk yield was lower in the FR group compared to the Ctrl group until the end of the experimental period (*p* ≤ 0.05; Table 5). In the Ctrl group, milk yield constantly decreased until the end of the experimental period (*p* ≤ 0.05). In the FR group, milk yield decreased from week 1 to week 3 (*p* ≤ 0.05), while milk yield increased from week 3 to week 5 (*p* ≤ 0.05). Fat, lactose, and protein percentages and SNF and EVL were not affected by either feed restriction or time postpartum (*p* > 0.05) in both the Ctrl and FR groups.

## 4. Discussion

In the next decades, dairy ruminants raised in tropical and arid regions will need to cope with more adverse climate conditions and longer periods characterized by droughts and pasture scarcity. These facts will have a direct impact on the health status and performance of dairy ruminants. Consequently, several studies have been performed to assess the consequences of feed restriction on health and performance in dairy cows [14,15], sheep [16,17,18], and goats [19,20,21]. To the best of our knowledge, the present study is the first investigating the effects of feed restriction during both prepartum and postpartum periods on BW, blood metabolites, and hormones as well as colostrum and milk yield and composition in fat-tailed dairy ewes.

In this study, the induced feed restriction reduced DMI in the FR group compared to the Ctrl group during both prepartum and postpartum periods. Despite the reduced DMI, no differences in BW were detected between groups during both prepartum and postpartum periods. Fat-tailed sheep are well known for being highly resilient to harsh environmental conditions, water scarcity, and seasonal weight loss. As described by Wilkes et al. [22], Damara sheep, a fat-tailed breed used for meat production, has a higher capacity to obtain more nutrients from low-quality diets than common sheep breeds such as Merino. In addition to the digestive capacity of this breed, another study from the same group also concluded that Damara sheep have higher tolerance to feed restriction and different fat mobilization regulation compared to Merino sheep [7]. As no differences in BW were detected in the present study, it seems that the animals in the FR group were able to adapt to the short-term feed restriction during both the prepartum and postpartum periods.

Metabolic status as well as hormonal regulation are two crucial factors involved in the mammary gland development during the dry period in sheep [23]. In addition, dairy ruminants around parturition are often under NEB due to the reduced feed intake capacity and the increased energy output [5]. Under this condition, large amounts of body reserves are mobilized to meet the energy requirements, which affect the concentrations of diverse metabolites and hormones in the bloodstream [24,25,26]. Due to these facts, prepartum and postpartum feed restrictions were expected to intensify NEB and consequently affect to a larger extend both the metabolic status and the hormonal regulation around parturition. In the present study, FFA and BHB were the only blood metabolites that increased in the FR group during both prepartum and postpartum periods. Moreover, blood TG concentrations were increased in the FR group postpartum. Increased FFA, BHB, and TG concentrations indicate a more intense fat mobilization in the FR group than the Ctrl group, which is in agreement with previous results observed in dairy sheep under feed restriction [27,28]. As described by Kalyesubula et al. [29], extensive fat mobilization decreases the concentration of VLDL, which increases the continuous accumulation of TG and cholesteryl esters in the hepatocytes. As a result of the fat accumulation, hepatocytes are damaged, and they release enzymes such as GOT, GPT, and LDH into the bloodstream [30]. As none of the liver enzymes (i.e., GOT, GPT, and LDH) were affected by either the prepartum or the postpartum feed restriction, it can be hypothesized that either the extend of fat mobilization observed in the FR group was moderate and had no negative consequences on the liver functionality, or the fat-tailed sheep has an exceptional capacity to mobilize fat without disturbing liver functionality. Although no differences in glucose concentrations were observed during the entire experimental period, feed restriction decreased insulin concentrations during both prepartum and postpartum periods. This might be related to the increased physiological responsiveness to circulating insulin caused by a reduced energy intake (i.e., carbohydrates). Under these conditions, reduced insulin concentrations in the FR group might trigger a similar physiological response to the one observed in the Ctrl group. Additionally, feed restriction caused increased GH concentrations during both prepartum and postpartum periods. As described in humans, states of either NEB or starvation result in increased GH resistance and increasing GH concentrations in the bloodstream [31]. According to diverse studies, increased GH during NEB is thought to preserve glucose homeostasis by antagonizing the systemic actions of insulin and promoting lipolysis and lipid oxidation [32,33]. This fact could explain the lack of differences in glucose concentrations between groups, as well as the increased FFA concentrations, and the decreased insulin concentrations observed in the FR group.

As showed by Ollier et al. [34] in dairy cows, reduced energy intake (i.e., dry hay) rapidly decreased circulating prolactin concentration compared to cows fed a normal late lactation diet. As described by these authors, reduced prolactin concentration caused a reduction in milk yield, which is in agreement with previous studies performed in dairy cows [35,36]. Despite the lack of literature about the effect of feed restriction on prolactin concentrations in fat-tailed dairy sheep, it seems that similar to dairy cows, prolactin concentrations decrease when these animals are under feed restriction, decreasing colostrum and milk yield. Prolactin is a key regulator of the mammary gland renewal and development as well as lactation [37]. The renewal of the mammary cells, which is crucial for next lactation, occurs during the dry period [38]. Changes due to feeding level might affect mammary gland reorganization and therefore have severe consequences in colostrogenesis and lactogenesis during this period. Colostrogenesis is defined as the prepartum transfer of components from the maternal circulation into mammary secretions, which starts several weeks before parturition and ceases abruptly immediately before parturition [39,40]. As described by Castro et al. [41], nutritional deficiencies in sheep and goats during the dry period have negative consequences on colostrum synthesis. Based on these facts, it seems that despite the FR group receiving the same diet as the Ctrl group at week −5 and week −1 relative to parturition, the induced feed restriction during weeks −4, −3, and −2 relative to parturition was sufficient to affect colostrogenesis, reducing colostrum yield. Furthermore, it seems that the reduced colostrum yield caused a relative up-concentration of the major components in colostrum, which could explain the increased fat, lactose, protein, and SNF percentages obtained in the FR group. Similarly, milk yield was also lower in the FR group than in the Ctrl group during the first five weeks postpartum, including week 1 and week 5 relative to parturition when the FR group received 100% of the diet offered to the Ctrl group. In agreement with these findings, several studies have described how the feed restriction during prepartum affects milk yield in diverse dairy sheep breeds such as Ghezel [18], Shropshire [42], and Santa Ines [43]. Even though fat-tailed sheep are well known for their ability to not decrease BW during periods of either feed restriction or pasture scarcity, it seems that feed restriction prepartum in fat-tailed dairy sheep affected the mammary gland reorganization and decreased colostrum yield as well as milk yield during the first five weeks postpartum.

## 5. Conclusions

The present study concludes that feed restriction in fat-tailed dairy sheep does not affect BW during both prepartum and postpartum periods. However, feed restrictions through prepartum and postpartum affect blood metabolites (i.e., free fatty acids, beta-hydroxybutyrate, and triglyceride concentrations) and hormones (i.e., insulin, growth hormone, and prolactin concentrations), which in turn decreases colostrum and milk yields postpartum.

## Figures and Tables

**Table 1:** Diet composition and calculated nutrient composition of diets fed ewes during both pre- and postpartum periods (dry matter basis).

	Diets	
	Dry Diet	Early Lactation Diet
Ingredients, DM %		
Alfalfa hay	26.7	39.0
Barley	34.9	39.0
Wheat straw	35.5	22.0
Chemical Composition ^1^		
DM ^2^, %	89.0	89.0
Calculated ME ^2^, Mcal/kg	2.13	2.23
CP ^2^, DM %	11.0	13.1
NDF ^2^, DM %	49.0	42.3
ADF ^2^, DM %	32.9	29.0
EE ^2^, DM %	2.10	2.31
Calcium, DM %	0.51	0.65
Phosphorous, DM %	0.24	0.27

^1^ Estimated using values obtained from the NRC [10]. ^2^ DM = Dry matter; ME = Metabolizable energy; CP = Crude protein; NDF = Neutral detergent fiber; ADF = Acid detergent fiber; EE = Ether extract.

**Table 2:** Plasma metabolites concentrations in the control (Ctrl; *n* = 10) and feed-restricted (FR; *n* = 10) groups prepartum (from week −5 to week −1) and postpartum (from week 1 to week 5). Least square means and standard error of the mean (SEM) are presented in this table.

Variables ^1^	Prepartum						Postpartum					
	Groups			Fixed Effects ^2^			Groups			Fixed Effects ^2^		
	Ctrl	FR	SEM	Feed Restriction	Time	F × T	Ctrl	FR	SEM	Feed Restriction	Time	F × T
Glucose, mg/dL	49.4	54.7	1.68	0.21	0.80	0.77	56.5	57.9	1.76	0.66	0.71	0.60
TP, g/dL	4.50	5.08	0.20	0.12	0.98	0.20	4.68	4.80	0.21	0.70	0.17	0.78
Urea, mg/dL	9.70	9.49	0.90	0.73	0.44	0.88	10.6	9.45	0.85	0.49	0.27	0.38
Albumin, g/dL	2.97	2.93	0.11	0.95	0.26	0.97	2.92	2.93	0.11	0.93	0.61	0.25
Creatinine, mg/dL	1.07	0.91	0.08	0.28	0.50	0.10	1.28	1.04	0.08	0.10	0.53	0.22
FFA, mg/dL	13.8	17.8	2.26	≤0.01	≤0.01	0.18	9.32	16.7	3.11	0.05	≤0.01	0.10
BHB, mg/dL	8.02	11.5	1.20	≤0.01	≤0.01	0.22	5.93	10.1	1.46	≤0.01	0.02	0.18
TG, mg/dL	62.5	63.0	0.64	0.58	0.23	0.40	61.5	64.4	0.99	0.04	0.01	0.24
Cholesterol, mg/dL	31.8	32.1	1.52	0.91	0.43	0.87	33.9	29.8	1.57	0.20	0.97	0.32
GOT, U/L	63.8	76.2	5.17	0.20	0.73	0.81	83.1	69.2	5.95	0.22	0.98	0.35
GPT, U/L	18.8	20.5	1.90	0.53	0.19	0.64	19.9	16.6	1.70	0.40	0.61	0.45
LDH, U/L	329	415	29.7	0.25	0.39	0.98	481	529	42.4	0.65	0.92	0.33

^1^ TP = Total Protein; FFA = Free fatty acids; BHB = Beta-hydroxybutyrate; TG = Triglycerides; GOT = Glutamic oxaloacetic transaminase; GPT = Glutamate–pyruvate transaminase; LDH = Lactate dehydrogenase. ^2^ F × T = Feed Restriction × Time interaction.

**Table 3:** Plasma hormones concentrations in the control (Ctrl; *n* = 10) and feed-restricted (FR; *n* = 10) groups prepartum (from week −5 to week −1) and postpartum (from week 1 to week 5). Least square means and standard error of the mean (SEM) are presented in this table.

	Prepartum						Postpartum					
Variables ^1^	Groups			Fixed Effects ^2^			Groups			Fixed Effects ^2^		
	Ctrl	FR	SEM	Feed Restriction	Time	F × T	Ctrl	FR	SEM	Feed Restriction	Time	F × T
Insulin, pmol/L	10.0	9.65	0.32	0.05	0.18	0.03	9.32	7.83	0.49	≤0.01	0.22	0.04
GH, ng/mL	0.48	0.58	0.02	≤0.01	≤0.01	≤0.01	0.75	0.83	0.03	≤0.01	≤0.01	≤0.01
PRL, ng/mL	115	106	3.92	≤0.01	≤0.01	≤0.01	132	86.1	4.61	≤0.01	≤0.01	≤0.01
P4, ng/mL	3.16	3.17	0.29	0.97	≤0.01	0.18	0.28	0.29	0.16	0.32	0.68	0.14

^1^ GH = Growth hormone; PRL = Prolactin; P4 = Progesterone. ^2^ F × T = Feed Restriction × Time interaction.

**Table 4:** Colostrum yield and composition in the control (Ctrl; *n* = 10) and feed restricted (FR; *n* = 10) groups. Least square means and standard error of the mean (SEM) are presented in this table.

Variables	Groups			Fixed Effects
	Ctrl	FR	SEM	Feed Restriction
Colostrum yield, kg	4.45	3.16	0.45	0.05
Fat, %	9.48	13.9	1.50	0.03
Lactose, %	13.1	14.9	0.87	0.04
Protein, %	9.52	10.7	0.58	0.05
SNF ^1^, %	26.9	30.1	1.60	0.04

^1^ SNF = Solids not fat.

**Table 5:** Milk yield and composition during the first 5 weeks of lactation in the control (Ctrl; *n* = 10) and feed-restricted (FR; *n* = 10) groups. Least square means and standard error of the mean (SEM) are presented in this table.

Variables	Groups			Fixed Effects ^2^		
	Ctrl	FR	SEM	Feed Restriction	Time	F × T
Milk yield, kg				≤0.01	≤0.001	≤0.001
week 1	1.28 ^a,^*	0.89 ^A^	0.09			
week 2	1.29 ^a,^*	0.50 ^B^	0.09			
week 3	1.19 ^a,b,^*	0.57 ^B,C^	0.08			
week 4	1.14 ^b,^*	0.66 ^C^	0.08			
week 5	1.02 ^b^	0.76 ^D^	0.13			
Fat, %				0.86	0.13	0.70
week 1	3.64	4.03	0.59			
week 2	3.27	3.82	0.47			
week 3	4.68	3.93	0.84			
week 4	4.22	4.58	0.51			
week 5	5.26	4.96	0.65			
Lactose, %				0.96	0.84	0.66
week 1	5.96	5.98	0.89			
week 2	5.89	6.00	1.63			
week 3	5.94	5.86	1.22			
week 4	5.94	5.95	1.95			
week 5	5.82	5.87	1.50			
Protein, %				0.78	0.80	0.81
week 1	4.07	3.99	1.22			
week 2	3.97	4.01	1.05			
week 3	3.99	3.95	0.77			
week 4	4.05	3.97	1.31			
week 5	3.88	3.92	0.99			
^1^ SNF, %				0.96	0.76	0.27
week 1	10.8	10.9	1.70			
week 2	10.7	11.1	1.14			
week 3	10.8	10.7	1.91			
week 4	10.9	10.8	1.77			
week 5	10.6	10.7	1.77			
EVL, Mcal/kg				0.59	0.22	0.57
week 1	0.82	0.86	0.04			
week 2	0.78	0.85	0.04			
week 3	0.86	0.84	0.04			
week 4	0.78	0.91	0.05			
week 5	0.96	0.94	0.06			

Lowercase superscripts (a,b) indicate significant differences (*p* ≤ 0.05) between time points within Ctrl group. Uppercase superscripts (A–D) indicate significant differences (*p* ≤ 0.05) between time points within FR group. * Indicates a significant difference (*p* ≤ 0.05) between experimental groups for a specific time point. ^1^ SNF = Solids not fat; EVL = Energy value of milk. ^2^ F × T = Feed Restriction × Time interaction.

## Data Availability

The data presented in this study are available on request from the corresponding author.
